# The relationship between posttraumatic stress symptoms and narrative structure among adolescent terrorist-attack survivors

**DOI:** 10.3402/ejpt.v7.29551

**Published:** 2016-03-16

**Authors:** Petra Filkuková, Tine K. Jensen, Gertrud Sofie Hafstad, Hanne Torvund Minde, Grete Dyb

**Affiliations:** 1 Norwegian Centre for Violence and Traumatic Stress Studies, Oslo, Norway; 2 Department of Psychology, University of Oslo, Oslo, Norway

**Keywords:** Posttraumatic stress symptoms, narrative organization, narrative fragmentation, internal events, external events

## Abstract

**Background:**

The structure of trauma narratives is considered to be related to posttraumatic stress symptomatology and thus the capacity to make a coherent narrative after stressful events is crucial for mental health.

**Objective:**

The aim of this study is to understand more of the relationship between narrative structure and posttraumatic stress symptoms (PTSS). More specifically, we investigated whether internal and external focus, organization, fragmentation, and length differed between two groups of adolescent survivors of a mass shooting, one group with low levels of PTSS and one group with high levels of PTSS.

**Method:**

The sample comprised 30 adolescents who survived the shooting at Utøya Island in Norway in 2011. They were interviewed 4–5 months after the shooting and provided a free narrative of the event. PTSS were assessed using the UCLA Posttraumatic Stress Disorder Reaction Index (PTSD-RI).

**Results:**

We found that survivors with high levels of PTSS described more external events and fewer internal events in their narratives compared with survivors with low levels of symptoms. The analysis also showed that especially narratives containing more descriptions of dialogue and fewer organized thoughts were related to higher levels of PTSS. The groups did not differ in levels of narrative fragmentation or in length of the narratives.

**Conclusion:**

Specific attributes of narrative structure proved to be related to the level of PTSS. On the basis of our results, we can recommend that practitioners focus especially on two elements of the trauma narratives, namely, the amount of external events, particularly dialogues, within the narrative and the number of organized thoughts. Participants with high levels of PTSS provided trauma narratives with low amount of organized (explanatory) thoughts accompanied by detailed descriptions of dialogues and actions, which is indicative for “here and now” quality of recall and a lack of trauma processing.

Every day, people experience unexpected and traumatizing events. When people encounter something unexpected, they tend to create narratives, which help them organize their experience and understand its meaning (Bruner, 1990; Pennebaker & Seagal, 1999; Tuval-Mashiach et al., 2004). Several scholars have accentuated individuals’ capacity to make a coherent narrative after stressful events as crucial for mental health (Foa, Molnar, & Cashman, 1995; Pennebaker & Seagal, 1999; Smyth, True, & Souto, 2001; Tuval-Mashiach et al., 2004). Translating feelings, sensations, and mental pictures into words helps people to organize and reflect on what has happened and may lead to less rumination (Follmer Greenhoot, Sun, Bunnell, Lindboe, & Lindboe, 2013) and helps diminish their reexperiencing of traumatic memories (Pennebaker & Seagal, 1999).

A narrative is a temporal structured story that links past and present together and helps us gain a sense of continuity (Nelson, 1999). According to Morgan (2000), “A narrative is like a thread that weaves events together, forming a story.” (pp. 5). The construction of meaning is considered vital for human development and coping, and a narrative contributes to meaning making when the “who,” “when,” “where,” and “what” of an event are woven together into a “why” (Aldwin, 2007; Fivush & Baker-Ward, 2005; Fivush, Edwards, & Mennuti-Washburn, 2003). The meaning of an event is thus often expressed through words that reflect the person's thoughts, feelings, and interpretations (Chase, 2005; Fivush & Nelson, 2006; Labov, 1997).

Several scholars have posited a close link between the organization of the trauma in an individual's memory and in his/her narrative and that posttraumatic stress disorder (PTSD) indicates poor processing of the trauma (Brewin, Dalgleish, & Joseph, 1996; Ehlers & Clark, 2000; Foa & Riggs, 1993). Traumatic memories are often disorganized because they are encoded under extreme stress (Foa & Riggs, 1993). Because of this stress, the trauma memory is often poorly elaborated and inadequately integrated into its context in time, place, and other memories (Ehlers & Clark, 2000). This results in disorganized trauma memories and possibly disorganized trauma narratives (Ehlers & Clark, 2000).

Based on predictions from cognitive models of PTSD (Brewin et al., 1996; Ehlers & Clark, 2000; Foa & Rothbaum, 1998), specific attributes of trauma narratives have been investigated (length, organization, fragmentation, sensory perceptions, negative emotions, etc.). A review article of these studies concluded that despite the elaborate theoretical basis, empirical findings on the relationship between narrative structure and PTSD are inconclusive (O'Kearney & Perrott, 2006).

One line of investigation has been the examination of the relationship between posttraumatic stress symptoms (PTSS) and narrative length. Theoretical accounts propose that trauma recall may increase when arousal decreases (Heuer & Reisberg, 1992). However, empirical findings on the relationship between trauma narrative length and symptom level are mixed. Whereas some studies have found a tendency toward a negative relationship between the level of PTSS and trauma narrative length (Foa et al., 1995; Jelinek et al., 2010), other studies have found a tendency in the opposite direction, indicating that individuals with high PTSS have longer trauma narratives (Gray & Lombardo, 2001). Some studies have not found any traces of a relationship at all (Halligan, Michael, Clark, & Ehlers, 2003; Jones, Harvey, & Brewin, 2007).

Other studies have investigated the relationship between narrative fragmentation and PTSD and have found that the narratives of highly traumatized individuals are more fragmented and disorganized (e.g., Halligan et al., 2003; Harvey & Bryant, 1999; Jones et al., 2007; Kenardy et al., 2007; Peterson & Biggs, 1998; Salmond et al., 2011). Jones et al. (2007) found that three aspects of narrative organization, namely, repetitions, non-consecutive chunks (utterance units that were out of order or were incongruous with each other) and overall coherence, predicted later PTSD severity among road traffic accident survivors. Similarly, Halligan et al. (2003) found that narrative disorganization measured 3 months after trauma predicted level of PTSD 6 months after trauma. These studies indicate that fragmented and disorganized narratives may be related to PTSD. However, other studies have not found this relationship (Gray & Lombardo, 2001; Rubin, 2011). O'Kearney and Perrott (2006) attributed the varying results to heterogeneity in terms of types of studies, operationalization, comparison groups, types of traumas, time passed since the trauma, and age and gender of the participants.

The degree of organized thoughts is assumed to be related to PTSD. High levels of organized thoughts may be indicative of good trauma processing and can be expressed by reasoning, planning, and reflections on why something happened (Foa et al., 1995). Organized thoughts can thus be connected to meaning making efforts, which are, according to Jerome Bruner (1990), the essence of narrative work. Some studies have found a relationship between level of organized thoughts and PTSD (Foa et al., 1995; Jelinek et al., 2010). Use of words such as “because,” “thus,” “therefore,” “realize,” “understand,” which commonly occur in organized thoughts, indicated good cognitive processing of trauma in studies by Mundorf and Paivio (2011) and Alvarez-Conrad, Zoellner, and Foa (2001).

In addition, the relationship between trauma narratives that contain many “internal events” such as thoughts and feelings, and narratives containing many “external events” such as descriptions of actions and dialogues have been examined (Foa et al., 1995; Van Minnen, Wessel, Dijkstra, & Roelofs, 2002). Foa et al. (1995) analyzed rape narratives at the beginning and at the end of prolonged exposure therapy, which involved repeatedly working with the trauma narrative. They found that from pre- to posttreatment, percentage of “internal events,” consisting of descriptions of thoughts and feelings, increased. In addition, there was a tendency toward a lower percentage of “external events,” consisting of descriptions of actions and dialogues, but these findings did not reach statistical significance. The researchers argued that the results may indicate that there is a shift toward more processing of feelings and that this shift is related to lower levels of PTSD. Furthermore, as the memory of the trauma becomes less frightening during therapy, it becomes less important for victims to remember “external” details of the event. It is also possible that as avoidance symptoms decline, victims may be less resistant to relating to their own feelings and thoughts.

Van Minnen et al. (2002) compared not only pre- and posttreatment narratives but also patients whose psychological health improved and did not improve in the course of the therapy. In their study, “internal events” encompassed descriptions of thoughts, feelings, and sensations (visual, auditory, tactile, olfactory, gustatory). “External events” consisted of descriptions of actions, dialogues, and details. Whereas the percentage of “internal events” increased after therapy, the percentage of “external events” and “disorganized thoughts” decreased. However, the only difference between improved and non-improved patients was that improved patients displayed a greater decrease in disorganized thoughts, and the authors argued that the changes in narratives are thus related to processing and elaboration of the narratives rather than to a reduction in symptom levels. As such, findings are somewhat mixed, and the theorized relationship between PTSD and narrative structure requires more thorough empirical investigation.

In the current study, we investigate the structure of trauma narratives on the sample of the adolescent survivors of the 2011 terrorist attacks in Norway. There have been a number of studies published on this sample (e.g., Aakvaag, Thoresen, Wentzel-Larsen, Røysamb, & Dyb, 2014; Dyb et al., 2013; Dyb, Jensen, Glad, Nygaard, & Thoresen, 2014; Hafstad, Dyb, Jensen, Steinberg, & Pynoos, 2014; Jensen, Thoresen, & Dyb, 2015; Thoresen, Jensen, Wentzel-Larsen, & Dyb, 2014), but all of them were analyzing data from questionnaires. None of the studies have so far focused on the analysis of the free narratives, where the participants described what happened during the terrorist attack.

## The 2011 Norway attacks

On July 22, 2011, there were two sequential terrorist attacks in Norway. First, a bomb exploded at the Government quarter in Oslo. Then, the attack continued with a spree shooting on Utøya Island, approximately 38 km from Oslo, where the Norwegian Labour Youth organization held a summer camp. The terrorist came to the island in a disguise; he was dressed as a policeman and claimed that he was sent to the island to secure the summer camp after the terrorist attack in Oslo. At the point when the terrorist arrived to Utøya, there were 564 people on the island, most of them adolescents. It took 1.5 h from the time the shooting started for the terrorist to be arrested. The island has an area of only 0.12 km^2^; thus, the possibilities for an individual to successfully hide for an extended period of time were very limited. Therefore, many adolescents decided to try to escape by swimming away from the island. Most swimmers were rescued from the lake by volunteers, who took out their boats when they heard shooting from the island. As a result of the attack on Utøya, 69 people died and many were injured.

## Aims of the study

The overall aim of this study is to gain a better understanding of the relationship between narrative structure and PTSS. More specifically, we aim to investigate whether internal and external focus of narratives, their organization, fragmentation, and length differ between two groups of adolescent survivors of a mass shooting, one group with low levels of PTSS and one group with high levels of PTSS.

Based on theory and literature, we hypothesize that the narratives of survivors with low levels of PTSS: 1) contain more internal events (descriptions of thoughts and feelings), 2) contain fewer external events (descriptions of actions and dialogues), 3) contain more organized thoughts, and 4) are less fragmented than narratives of survivors with high levels of PTSS. Given the mixed empirical findings on the relationship between symptom level and trauma narrative length, we do not set a hypothesis in any particular direction.

## Method

### Participants and procedure

The police registered 495 survivors of the terrorist attack on Utøya Island. Three months after the terrorist attack, the 490 survivors who were at least 13 years of age were sent postal invitations to participate and were subsequently contacted by phone. One hundred and sixty-five survivors could not be reached by phone or declined to participate, whereas 325 (66.3%) survivors were interviewed face-to-face; most of the interviews occurred at the participants’ homes. There were no significant differences in gender or age between participants and non-participants. Most interviews (95.4%) were conducted in November and December 2011. The interview was semi-structured and was performed by health personnel. Prior to the interviews, the interviewers were trained regarding interviewing techniques for traumatized populations. Interviewers asked participants to freely describe what they experienced during the terrorist attack. The accounts were audio recorded and subsequently transcribed verbatim.

To minimize the role of age on the analyses, we limited the sample in this paper to those who were aged 16–20 years at the time of the terrorist attack. The majority of the total sample (226 participants, that is, 69.5% of the total of 325 participants) fell into this age group. Subsequently, we ranked all the 226 participants in the given age group according to their individual scores of posttraumatic stress reactions measured by UCLA PTSD Reaction Index (PTSD-RI) (see a detailed description of the scale below). Our aim was to compare two extreme groups of participants, that is, one group with very high and one group with very low scores of posttraumatic stress reactions. We selected 16 women and 14 men from 20 participants with the highest and 20 participants with the lowest posttraumatic stress reaction scores. The selection of these 30 participants was guided by the aim to make the high and the low PTSS score groups gender-balanced because gender differences in trauma narratives have been found in previous studies (e.g., Bohanek & Fivush, 2010). Each group had 15 participants (high PTSS score group: *M*_PTSS_=48.34, *SD*=2.48; low PTSS score group: *M*_PTSS_=6.02; *SD*=1.92); the mean age of the analyzed sample was 17.8 years.

### Measures

#### Posttraumatic stress reactions

Posttraumatic stress reactions over the past month were measured using the UCLA PTSD-RI (Pynoos, Rodriguez, Steinberg, Stuber, & Frederick, 1998; Steinberg, Brymer, Decker, & Pynoos, 2004). The PTSD-RI is a 20-item scale in which responses are recorded on a five-point scale, ranging from zero (never) to four (most of the time). Three items have two alternative formulations, and the highest score is applied to calculate the total score. Hence, 17 items make up the total symptom scale score, corresponding to the PTSD criteria of the Diagnostic and Statistical Manual of Mental Disorders, fourth edition (DSM–IV). Five items describe re-experiencing, seven items describe avoidance and five items describe increased arousal. In the present study, mean scores were computed and applied in the analyses.

### Narrative coding system

To code the narratives, we used the full version of the manual developed by Foa, DiSavino, and Turk (n.d.). The advantage of using this manual is its extensiveness as it covers the whole text of the narrative and was successfully used before.

Most of the 21 categories are related to the content of the utterance, but some refer only to formal aspects. The separate categories are grouped into seven large overarching categories, namely, “thoughts,” “feelings,” “actions,” “dialogues,” “sensations,” “details,” and “non-functional utterances.”

“Thoughts” consist of four categories: 1) “organized thoughts,” referring to planning, reasoning, hypothesis setting, decision making, and realizing; 2) “disorganized thoughts,” involving confusion and uncertainty; 3) “desperate thoughts,” implying that all coping strategies were unavailable; and 4) “unfinished thoughts,” that is, a sentence attempted but not completed.

“Feelings” are divided into 1) “positive feelings,” 2) “negative feelings,” and 3) “adaptive angry feelings.”

“Actions” consist of five categories: 1) “action-self,” referring to the narrator's own behavior; 2) “action-other,” involving actions of other people than the narrator and the perpetrator; 3) “action-perpetrator-threat”; 4) “action-perpetrator-non-threat”; and 5) “action-joint,” involving actions that the narrator pursued with the perpetrator.

“Dialogues” consist of five categories: 1) “dialogue-self-other,” referring to the narrator's communication toward people other than the perpetrator; 2) “dialogue-self-perpetrator,” referring to the narrator's communication with the perpetrator; 3) “dialogue-perpetrator-threat,” involving threatening communication made by the perpetrator to the victim; 4) “dialogue-perpetrator-non-threat”; and 5) “dialogue-other,” referring to descriptions of verbalization made by persons other than the narrator or the perpetrator.

The category “sensations” refers to visual, auditory, tactile, olfactory, and gustatory perceptions. “Details” involve passive descriptions not falling into any of the abovementioned categories; Foa et al. (1995) also called these descriptions “miscellaneous utterance category.”

In addition to these content-based categories, Foa et al. (n.d.) categorized “non-functional utterances” into 1) “repetitions,” 2) “speech fillers,” and 3) “unfinished thoughts.” Unfinished thoughts hence have a double categorization, which was followed in the analyses of both Foa et al. (1995) and Van Minnen et al. (2002).

The coding should cover the whole text of the trauma narrative; only sentences involving responses to direct questions posed by the interviewer are labeled as “not coded.” However, the categories are not designed as completely exclusive; in the case of category overlap, a priority list should be applied (Foa et al., 1995).

In line with Foa et al. (1995), the beginning of the trauma narrative was defined as the first realization of danger and the end was defined as the end of the threat. In our case, the end referred to the moment when the survivors reached land. This part was present in all the interviews. Some participants also provided descriptions of hours before the attack and hours, days and even weeks after they reached safety; this part of the narratives was not included in the analysis.

The manual with a coding guide (Foa et al., n.d.) was attained by Edna Foa with the permission to use it for the study. The whole research team read the comprehensive manual and discussed the coding in meetings. Coding was trained on a trauma narrative not included in the current study; dilemmas in coding were resolved by consensus. Then two raters (the first and fourth author) coded five narratives simultaneously. The trauma narratives were uploaded in the program NVivo, where raters labeled the text by 21 categories from the manual by Foa et al. (n.d.) described above. Inter-rater agreement, as computed by the NVivo program, was between 88.7 and 100% for separate categories. NVivo does not provide a percentage agreement for the whole text. However, when all categories were taken together (regardless of their frequency in the text), the overall agreement was 98.3%. In group meetings with the coauthors the coded interviews were compared and discussed. The two raters worked then independently on coding of the remaining narratives.

The NVivo program helped to organize thousands of coding units and provided information on what percentage of the text of each trauma narrative belonged to each category. We used percentage (e.g., percentage of sensation–utterances in the text) instead of raw numbers (e.g., number of sensation–utterances in the text) so that interviews of different lengths would be weighted equally. The two groups (high PTSS score versus low PTSS score) were subsequently compared via *t* test in the SPSS program.

## Results

We found a tendency toward a higher percentage of “internal events” (“thoughts” and “feelings”) among participants with a low PTSS score, but the result did not reach statistical significance; *t*(28)=−1.85, *p*=0.075. A closer analysis revealed that the tendency was powered by “thoughts,” particularly “organized thoughts,” that is, utterances involving attempts to understand what is happening, such as planning, reasoning, hypothesis setting, decision making, realizing, etc.; *t*(28)=−2.50, *p*=0.022. There was no difference in the percentage of “feelings” between participants with high and low PTSS scores; *t*(28)=0.404, *p*=0.689.

In line with our hypothesis, narratives of participants with low PTSS scores contained fewer descriptions of “external events” (“actions” and “dialogues”) compared with the narratives of survivors with high levels of PTSS; *t*(28)=3.19, *p*=0.004. The detailed analysis revealed that survivors with high PTSS scores reported significantly more own dialogues with others during the attack (*t*(28)=3.60, *p*=0.001) and more frequently mentioned the communication of others (*t*(28)=2.34, *p*=0.027). The results from the analyses of the narrative structure in the two groups of survivors are displayed in Table 1.

**Table 1:** Comparison between low-PTSS and high-PTSS participants: separate subcategories and total category scores

		Low PTSS (*N*=15)		High PTSS (*N*=15)		
				
Categories		*M*	*SD*	*M*	*SD*	*p*
Internal events	Thoughts	24.81	9.03	19.05	4.81	0.041
	Organized thoughts	15.62	7.74	10.28	2.93	0.022
	Disorganized/confused thoughts	2.91	2.43	3.85	2.78	0.330
	Desperate thoughts	0.13	0.31	0.54	0.98	0.142
	Unfinished thoughts	6.14	4.64	4.37	2.30	0.197
	Feelings	2.24	2.38	2.54	1.57	0.689
	Positive feelings	0.69	1.27	0.47	0.95	0.591
	Negative feelings	1.37	1.28	2.00	1.25	0.185
	Adaptive angry feelings	0.19	0.49	0.08	0.22	0.441
	**Total**	**27.05**	**10.06**	**21.59**	**5.48**	**0.075**
External events	Actions	23.83	9.20	29.36	9.12	0.109
	Action-self	13.62	6.95	14.23	3.70	0.766
	Action-other	6.94	3.66	10.52	5.19	0.037
	Action-perpetrator-threat	2.52	3.22	3.96	3.49	0.249
	Action-perpetrator-non-threat	0.75	1.22	0.65	0.60	0.765
	Dialogues	5.47	3.86	12.52	6.39	0.001
	Dialogue-self-other	1.84	2.04	5.06	2.80	0.001
	Dialogue-other	3.47	3.40	6.92	4.59	0.027
	Dialogue-perpetrator-threat	0.03	0.09	0.15	0.59	0.439
	Dialogue-perpetrator-non-threat	0.12	0.45	0.17	0.48	0.770
	Dialogue-self-perpetrator	0.01	0.03	0.21	0.82	0.351
	**Total**	**29.30**	**8.30**	**41.87**	**12.84**	**0.004**
Fragmentation	Repetitions	2.91	2.34	3.32	1.82	0.597
	Speech fillers	1.24	1.38	1.22	1.36	0.973
	Unfinished thoughts	6.14	4.64	4.37	2.30	0.197
	**Total**	**10.30**	**6.44**	**8.92**	**3.61**	**0.476**
Other	Sensations	4.71	3.49	5.01	2.41	0.783
	Details	28.31	4.07	22.43	9.18	0.035

*Note*: “Not coded” is not listed in the table. “Action-joint,” referring to actions that the interviewee did jointly with the perpetrator, had a zero occurrence; thus, it is not listed in the table.

The level of “fragmentation” in the narratives, measured by the mean percentage of “repetitions,” “speech fillers,” and “unfinished thoughts,” was not significantly different between groups (participants with low and high PTSS scores; *t*(28)= −0.72, *p*=0.476).

The word length of the trauma narratives of survivors with high levels of PTSD (*M*=2206.7, *SD*=1264.7) compared to those with low PTSD levels (*M*=1780.8, *SD*=1278.6) was not significantly different; *t*(28)=0.917, *p*=0.367.

Two out of Foa et al.'s seven large overarching categories do not belong to any of the three sum scores of interest categories, namely, “sensations” and “details.” In the current study, these categories were categorized as “other” (Table 1). We found that the narratives of participants with low PTSS scores had a higher prevalence of “details” than those of participants with high PTSS scores (*t*(28)=−2.27, *p*=0.035). Details involve descriptions and elaborations, actions not taken, and actions described in passive voice (“I was bruised,” “I was taken to the car”) (Foa et al., n.d.).

## Discussion

The present study explored how narrative organization and content related to PTSS in a sample of adolescent survivors of a mass shooting. We found that survivors with high levels of PTSS described more external events and fewer internal events in their narratives, compared to those with low levels of symptoms. The groups did not differ in terms of levels of narrative fragmentation or length of the narratives. In our study, we found parallels between participants with high PTSS scores and Foa et al.'s patients before therapy and between participants with low PTSS scores and patients after therapy. These similarities include a higher prevalence of actions and dialogues (“external events”) and a lower prevalence of organized thoughts among participants with high PTSS scores. This parallel is interesting in light of the fact that Foa et al. attributed the narrative changes to the therapist's instruction to focus on one's thoughts and feelings during repeated reliving and retelling of the trauma. Van Minnen et al. (2002) found a nearly identical pattern of results in patients who improved and did not improve during the therapy; thus, they did not attribute the narrative changes to a beneficial effect of the treatment (decrease in PTSS) but to a general effect of repeatedly telling the story. Our study contributes to the discussion on the relationship between PTSS level and narrative structure because it compared participants with high and low levels of PTSS beyond the typical pre- versus post-therapy comparison. Our results suggest that external and internal events may be important indicators of PTSS level.

Our findings correspond with Ehlers and Clark's (2000) assumption that people with high PTSS scores have less organized trauma memories and more “here and now” quality of recall. Participants with low PTSS scores seem to view the trauma from a more distant viewpoint, using more details and explanatory utterances (organized thoughts) and fewer vivid descriptions, such as use of direct speech within the narrative. It is important to note that the differences in narrative structure between the two groups of participants were not related to different lengths of trauma narratives.

Contrary to Ehlers and Clark's assumptions, however, we did not find differences in fragmentation between participants with high and low PTSS scores. This result replicates the findings of Foa et al. (1995) and Van Minnen et al. (2002). In their review article, O'Kearney and Perrott (2006) concluded that definitions of fragmentation and organization are inconsistent across studies. They claimed that there is no easy way to objectively measure disorganization because it can encompass semantic disorganization (cognitive uncertainty, for instance, “I do not remember”), syntactic disorganization (disorganized sentence structure), temporal disorganization, and general difficulty in reading the interview transcript. Together with studies by Foa et al. (1995) and Van Minnen et al. (2002), also our study suggests that there is no difference in fragmentation between narratives of participants with high and low levels of PTSS when fragmentation is defined by the amount of repetitions, unfinished thoughts, and speech fillers in the trauma narrative.

Our study contributes to the growing body of knowledge that indicates no significant difference in the length of trauma narratives between participants with high and low levels of PTSS (e.g., Halligan et al., 2003; Jones et al., 2007) and that the non-significant tendencies going in opposite directions that have been found in some studies might be explained by random fluctuations. Despite the strengths of this study, there are some limitations that need to be addressed.

First, the sample is rather small, and non-significant findings should be interpreted with that in mind. Although the size of our sample was larger than that of any of the previous studies applying the same narrative coding manual (Foa et al., 1995; Van Minnen et al., 2002), the detection of smaller differences between the groups may be compromised due to limited statistical power. Second, the manual composed by Foa et al. operates on a micro-level as it is used for the categorization of very small text units (few words per one utterance unit). It does not contain a score related to how logically each sentence is connected to the following one or whether the whole narrative is comprehensible and chronologically organized. Third, it should be noted that the survivors of a terrorist attack with the highest probability repeatedly talked about their experience with others prior to meeting the researchers 4–5 months after the terrorist attack. This might have helped them to make the narrative more fluent and less fragmented in comparison with people in other studies who were only accepted to an emergency department or those who held secret the fact that they were victims of sexual violence (e.g., Foa et al., 1995; Peterson & Biggs, 1998).

In a clinical setting, it is not feasible to conduct a detailed quantitative analysis of trauma narratives. On the basis of our results, we can recommend that practitioners focus especially on two elements of the narratives, namely, the amount of dialogues within the narrative and the number of organized thoughts. Participants who still vividly reported what was said (often in a form of a direct speech) 4–5 months after the attack were among those with the highest PTSS scores. By contrast, low PTSS scores were associated with a high proportion of “organized thoughts,” which contained reasons for the participant's actions and, hence, made the trauma experience understandable to the listener on a more complex level.

In line with many therapy models (e.g., prolonged exposure therapy, narrative exposure therapy, cognitive behavioral therapy for PTSD, cognitive processing therapy, and TF-CBT) we recommend that clinicians help patients to elicit a trauma narrative and help them expand on the narrative by introducing thoughts and feelings that may contribute to meaning making and to shifting the focus from external events to internal events. This may help them process the event from something scary that happened in the past to just a memory that does not elicit fear.

It would be interesting for further studies to focus on particular themes of trauma narratives, which could have a relationship with PTSS (for instance, one's conviction of one's own survival versus death during the trauma). By limiting the analysis to only a few content elements, it would be feasible to analyze a larger sample of interviews.

We find it fruitful to focus attention on the amount of dialogue within a narrative, as dialogue seems to be positively related to the level of PTSS and may indicate the “here and now” quality of the recall several months after the trauma. Thus far, dialogue within the trauma narratives has only been studied by Foa et al. (1995) and Van Minnen et al. (2002). A closer examination could reveal whether the dialogue reported via direct speech (as opposed to indirect speech) has an even stronger relationship with high PTSS levels.

All in all, our study suggests that the level of PTSS is associated with specific attributes of narrative structure and more studies on the topic seem fruitful. Trauma narratives with large amount of vivid descriptions of actions and dialogues accompanied by low amount of organized (explanatory) thoughts should call for closer therapeutic attention, as they may be indicative of high level of PTSS.
